# Host Competence and Helicase Activity Differences Exhibited by West Nile Viral Variants Expressing NS3-249 Amino Acid Polymorphisms

**DOI:** 10.1371/journal.pone.0100802

**Published:** 2014-06-27

**Authors:** Stanley A. Langevin, Richard A. Bowen, William K. Reisen, Christy C. Andrade, Wanichaya N. Ramey, Payal D. Maharaj, Michael Anishchenko, Joan L. Kenney, Nisha K. Duggal, Hannah Romo, Aloke Kumar Bera, Todd A. Sanders, Angela Bosco-Lauth, Janet L. Smith, Richard Kuhn, Aaron C. Brault

**Affiliations:** 1 Center for Vectorborne Diseases and Department of Pathology, Microbiology and Immunology, School of Veterinary Medicine, University of California, Davis, California, United States of America; 2 Department of Biomedical Sciences, Colorado State University, Fort Collins, Colorado, United States of America; 3 Division of Vector-Borne Diseases, Centers for Disease Control and Prevention, Fort Collins, Colorado, United States of America; 4 Department of Biological Sciences, Purdue University, West Lafayette, Indiana, United States of America; 5 United States Fish and Wildlife Service, Portland, Oregon, United States of America; 6 Department of Biological Chemistry, University of Michigan, Ann Arbor, Michigan, United States of America; University of Texas Medical Branch, United States of America

## Abstract

A single helicase amino acid substitution, NS3-T249P, has been shown to increase viremia magnitude/mortality in American crows (AMCRs) following West Nile virus (WNV) infection. Lineage/intra-lineage geographic variants exhibit consistent amino acid polymorphisms at this locus; however, the majority of WNV isolates associated with recent outbreaks reported worldwide have a proline at the NS3-249 residue. In order to evaluate the impact of NS3-249 variants on avian and mammalian virulence, multiple amino acid substitutions were engineered into a WNV infectious cDNA (NY99; NS3-249P) and the resulting viruses inoculated into AMCRs, house sparrows (HOSPs) and mice. Differential viremia profiles were observed between mutant viruses in the two bird species; however, the NS3-249P virus produced the highest mean peak viral loads in both avian models. In contrast, this avian modulating virulence determinant had no effect on LD_50_ or the neurovirulence phenotype in the murine model. Recombinant helicase proteins demonstrated variable helicase and ATPase activities; however, differences did not correlate with avian or murine viremia phenotypes. These *in vitro* and *in vivo* data indicate that avian-specific phenotypes are modulated by critical viral-host protein interactions involving the NS3-249 residue that directly influence transmission efficiency and therefore the magnitude of WNV epizootics in nature.

## Introduction

Since 1999, WNV has expanded its geographic range to include both continents of the Western hemisphere and the Caribbean islands, with recent WNV activity documented on every major continent except Antarctica [1]–[8]. One hallmark feature associated with epizootic transmission events in North America has been avian mortality, predominantly in corvid species such as the American crow (AMCR; *Corvus brachyrhynchos*) [9]–[11]. Consequently, WNV dead bird surveillance programs have allowed, for the first time, a detailed molecular characterization of different geographic isolates as WNV expanded across vast ecological habitats in North America [12], [13]. Results of these studies have shown that WNV is genetically stable with maximum genetic variation of 0.2% at the amino acid level among isolates made during its trans-continental spread between 1999–2004 [13]. During this same time, WNV was responsible for large epizootics resulting in marked declines in highly susceptible avian populations[14]–[17].

**Figure 1:**
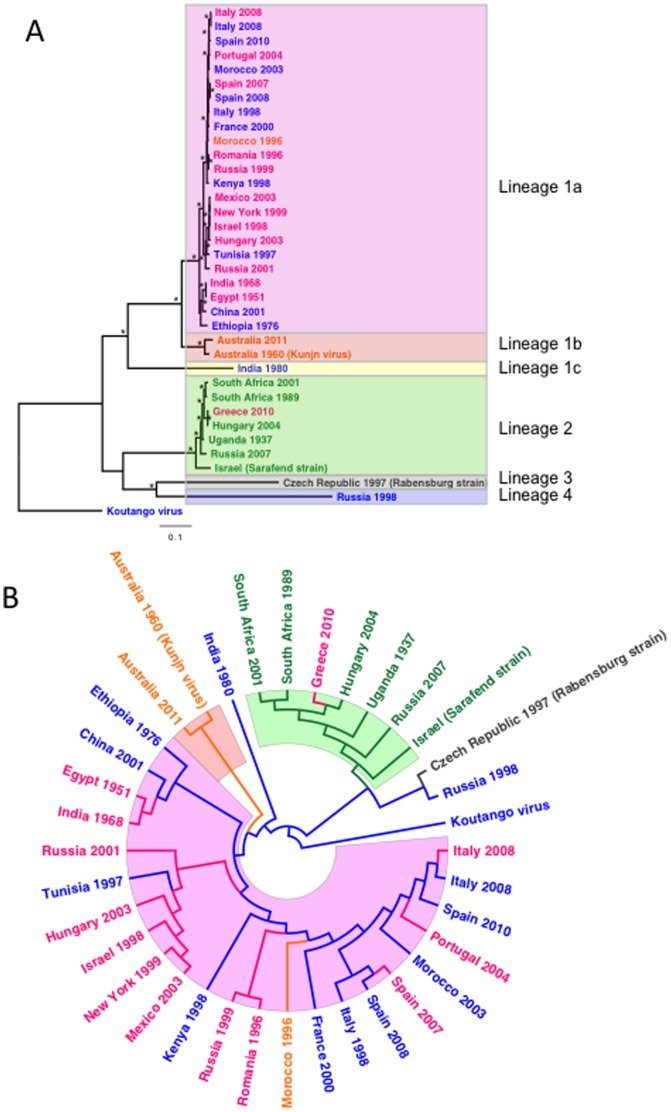
Phylogenetic analyses of genomic WNV strains. (A) Phylogenetic tree of the coding region of 36 WNV strains, constructed using Bayesian analysis (B) Maximum likelihood phylogenetic tree. Isolates are colored according to the amino acid at position 249 in NS3. Magenta  =  Pro; Blue  =  Thr; Green  =  His; Orange  =  Ala; Black  =  Asn. Asterisks represent nodes supported by at least 95% posterior probability. Clades are highlighted by WNV lineage. Pink  =  Lineage 1a; Orange  =  Lineage 1b; Yellow  =  Lineage 1c; Green  =  Lineage 2; Gray  =  Lineage 3; Blue  =  Lineage 4.

Previous studies have demonstrated the role of birds in the WNV transmission cycle as a mechanism for restricting viral genetic diversity through the effects of negative selection [18]. In contrast, a substitution in the WNV genome (NS3-T249P), associated with increased viremia production and mortality in AMCRs, has been shown to be under strong positive selective pressure, indicating that avian hosts can drive adaptive evolution in addition to serving as an important source of purifying selection [19]. A threonine (Thr) to proline (Pro) substitution has occurred at this locus on at least three independent occasions between lineage 1a WNVs, preceding human WNV outbreaks in Egypt (1950), Romania and Russia (1996) and Israel (1997–98) (Fig. 1). Considerable intra- and inter-lineage genetic heterogeneity has been observed at this helicase domain locus. For instance, Kunjin viruses (lineage 1b) have an alanine (Ala), lineage 2 WNVs are associated with histidine (His) at this locus [20] and lineage 3 [21] exhibit an asparagine (Asn), whereas many lineage 1 and the single representative lineage 4 WNV strain [21], [22] have a Thr at NS3-249 (Table 1, Fig. 1). Furthermore, a large outbreak of human encephalitis in Greece in 2010 has been associated with a lineage 2 WNV exhibiting a His to Pro substitution at this locus [23], demonstrating the selective importance of this site in alternative lineage WNVs as well. Interestingly, the H249P Greek lineage 2 WNV isolate has the closest genetic identity to a 2004 lineage 2 isolate from a goshawk that has been associated with avian virulence [24], indicating the potential selective role of avian replication/virulence for driving evolution at this genetic position. Furthermore, AMCRs inoculated with chimeric viruses generated between AMCR virulent (NY99) and non-virulent (KN3829) WNV strains [25] have demonstrated reversion at this locus from a 249T residue to variable amino acids including an aspartic acid mutation (249D), serving as additional evidence of the strong selective pressures exerted at this position.

**Table 1:** Genetic and geographic distribution of NS3-249 polymorphisms

WN/IC NS3-249 mutant	Nucleotide identity	Amino acid a	Reference strains with same NS3-249 identity b	Phylogenetic grouping
WN/IC-249P	CCC	Pro	North American 1999-, Romania 1996, Russia 1998, Egypt 1951, Greece 2010 (2)	Lineage 1a/2
WN/IC-249T	ACC	Thr	Ethiopia 1976, Kenya 1998, Tunisia 1997, Russia 1998 (4)	Lineage 1a/4
WN/IC-249A	GCC	Ala	Morocco 1996 (1a), Kunjin 1960- (1b)	Lineage 1a/1b
WN/IC-249H	CAC	His	Uganda 1937, South Africa 1989, Hungary 2004	Lineage 2
WN/IC-249N	AAC	Asn	Rabensberg (Czech Republic) 1997	Lineage 3
WN/IC-249D	GAC	Asp	Reverting mutation identified with chimeric infections in AMCRs.	NA-experimental mutation

a Pro: Proline; Thr: Threonine; Ala: Alanine; His: Histidine; Asn: Asparagine; Asp: Aspartic acid.

b Reference strains and respective lineage groupings that contain a particular amino acid at the NS3-249 position. See Fig. 1a-b. phylogenetic trees for a complete list of representative isolates (numbers in parentheses represent WNV lineage).

The existence of multiple natural (Ala, His, Pro, Thr and Asn) and experimentally derived (Asp) genetic variants at the NS3-249 locus, coupled with the finding that this residue imparts higher viremia potential in an avian host, highlight the potential for strong selective pressures being exerted on the NS3-249 locus. The emergence of lineage 1a and 2 WNVs incorporating a Pro residue at this site has served to support the hypothesis that this site could modulate the avian viremia capacity of various WNV lineage viruses and potentially dictate the epizootic emergence of these viruses. Recombinant viruses were generated in which five NS3-249 amino acids (Ala, Thr, His, Asn and Asp) were interchanged with the Pro of a lineage 1a cDNA clone and inoculated into AMCR and house sparrows (HOSPs) in order to assess the virulence potential in two reservoir avian species. In addition, we evaluated the effect of this WNV NS3-249 residue on mammalian virogenesis, by assessing the LD_50_ of 4 NS3-249 mutants in mice. Previous *in vitro* studies have demonstrated differences in temperature sensitivity among closely related WNV strains in mammalian and avian cell lines [26]. As such, the impact of NS3-249 amino acid substitutions on WNV growth exposed to elevated temperatures (37°C vs. 44°C) was also evaluated herein in an avian cell line. The two primary functions of the NS3 helicase are to separate viral RNA duplexes produced during viral RNA replication and to hydrolyze ATP to catalyze this enzymatic function [27]. Therefore, *in vitro* helicase bioassays and ATPase assays were performed to compare functional activity between the WNV NS3 helicase proteins expressing alternative NS3-249 amino acid residues.

## Results

### Sequence analyses

**Figure 2:**
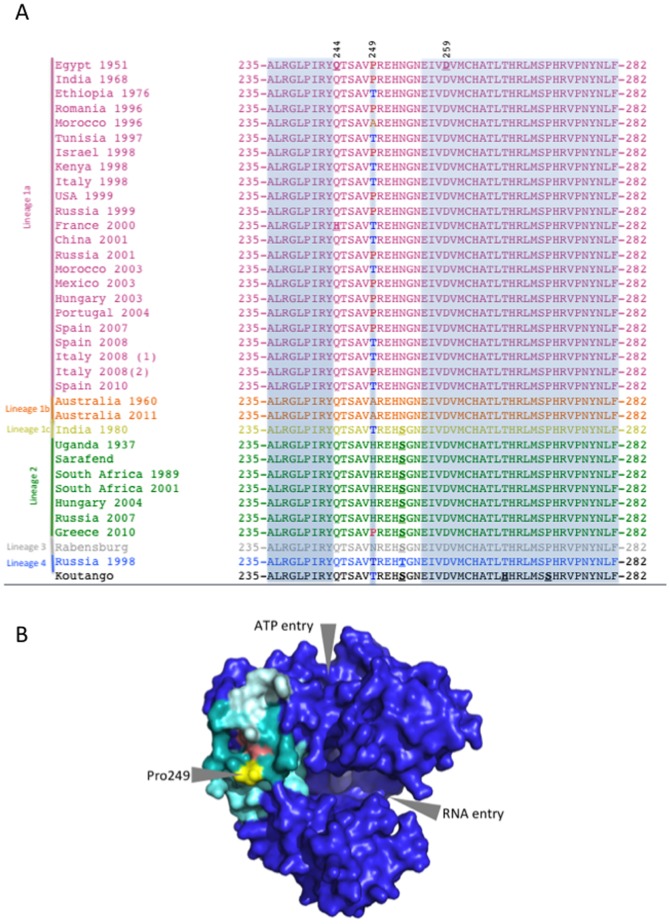
Sequence alignment of NS3-235–282 and helicase structure. (A) Alignment of 36 WNV strains between NS3 aa positions 235–282. The NS3-249 locus is indicated and the amino acid identities colored according to the same color scheme as depicted in panels A and B. Shaded areas correspond to aa 235–243 and aa 256–282, and emboldened and underlined text highlight genetic differences as well as sites of compensatory mutations (NS3-244 and NS3-259). Panel (B) Surface image depiction of the WNV helicase. Arrows depict RNA-entry site, ATP hydrolysis site and NS3-249 (yellow). Other substitutions identified in this study (salmon) include Q244H just above Pro249 and D259E just behind Q244. The peptides surrounding Pro249 include amino acids 256–282 (light cyan), 243–254 (dark cyan) and 235–243 (medium cyan).

In order to assess the extent of intra- and inter-lineage incorporation of the NS3-249 proline mutation, Bayesian (Fig. 1a) and Maximum likelihood (Fig. 1b) phylogenies of WNV strains from lineages 1–4 were constructed and the amino acid identity at position NS3-249 for individual isolates color-coded on the resulting phylogeny. In both analyses, results demonstrated the threonine (blue) to occupy the ancestral position at basal nodes and within this phylogeny, an NS3-249 mutation from a Thr to Pro, Ala, His or Asn has been demonstrated on at least twelve independent occasions. Eight of these mutations led to a NS3-249P (red/magenta) at this position, with seven of these NS3-T249P mutations having occurred within lineage 1a strains, and one having occurred in a lineage 2 strain (Greece 2010) (Fig. 2a)[23]. Such hypervariability at a particular locus was consistent with positive selection at this site as was previously reported [19]. To confirm this, we performed a Bayesian test to estimate the non-synonymous to synonymous substitution rate (dN/dS) of individual sites across all WNV lineages. Again, there was strong evidence for positive selection acting on the NS3-249 site (dN/dS  = 7.5, *p*<0.05). No other sites were identified in this analysis for positive selection. The NS3-249 residue is predicted to lie at the apex of a hydrophobic loop (Fig. 2b) and an alignment of the WNVs utilized for the phylogenetic analyses indicate complete conservation of the residues (aa 243–252; Fig. 2a) that form the loop with the exception of the NS3-249. These data suggest that a proline at NS3-249 may be adaptive in multiple WNV genetic backgrounds.

### Generation of recombinant WNVs

Recombinant viruses containing polymorphisms at the NS3-249 site were successfully generated following transfection with *in vitro* transcribed ligation products. Cytopathic effects were observed in all viral cultures within 3–4 days post-transfection. Prior to use for *in vitro* and *in vivo* phenotypic assessments, the complete genomes of all rescued viruses (NS3-249A, NS3-249D, NS3-249H, NS3-249P, NS3-249T, NS3-249N) were sequenced. When compared to the parental WN/IC-P991 virus, complete genomic sequencing of all rescued mutants demonstrated that the only variation occurred at the desired NS3-249 loci for each of the mutants.

### WN/IC NS3-249 point mutant plaque phenotypes and growth kinetics in a mammalian (Vero) cell line

**Figure 3:**
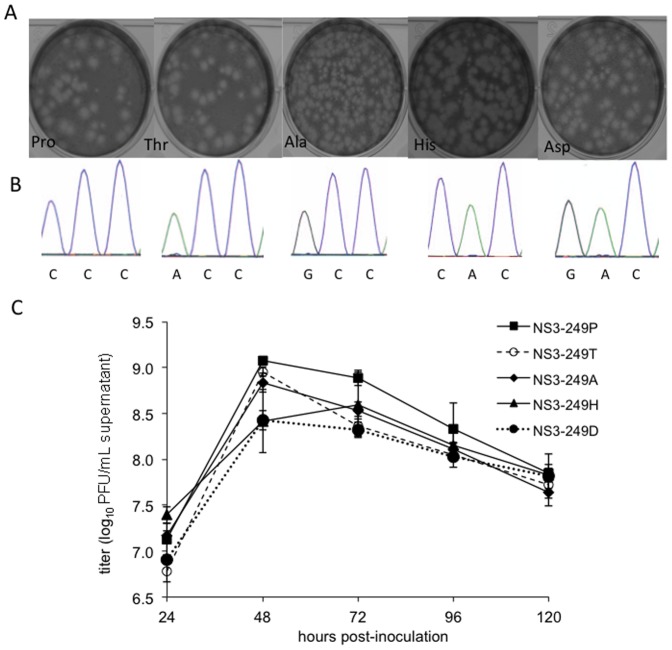
Phenotypic and genetic characterization of rescued WN/IC NS3-249 mutants. (A) WN/IC NS3-249 point mutant plaque phenotypes in a mammalian (Vero) cell line. Plaque diameters were determined to be 1.8±0.3 mm (249P), 1.7±0.4 mm (249T), 1.4±0.3 mm (249A), 1.6±0.3 mm (249H), 1.3±0.2 mm (249D) and 1.9±0.5 mm (249N; not shown). (B) Chromatogram depicting the sequence identity of the NS3-249 loci (genomic position 5456-5458) following generation of the recombinant viruses. (C) WN/IC NS3-249 point mutant growth profiles in a mammalian (Vero) cell line. Cells were inoculated at an MOI of 0.1. Bars represent standard deviation from the mean.

In order to assess the impact of introducing alternative NS3-249 amino acids into the WN/IC NY99 backbone on the growth kinetics in mammalian cells, we examined plaque morphology on Vero cells (Fig. 3a), assessed genetic stability at the modified NS3-249 loci (Fig. 3b), and quantified infectious virus as plaque forming units (PFU) from Vero cell culture supernatant collected daily for 5 dpi (Fig. 3c). Mean plaque diameters ranged between 1.9 to 1.3 mm at 3 dpi; plaques that were significantly smaller than the NS3-249P were identified for the 249A (p<0.01) and 249D mutants (p<0.01) (Fig. 3a, legend). No mixed genetic populations (Fig. 3b) were observed following propagation of the rescued mutants on Vero cells. During the first 24 hours, the WN/IC NS3-249D and NS3-249T generated titers that were at least 2-fold lower than the NS3-249A, NS3-249H, and NS3-249P viruses (p<0.05). There were no significant differences in mean peak viral titers between the NS3-249A, NS3-249H, and NS3-249T mutants when compared to the NS3-249P virus (p>0.1). In contrast, the WN/IC NS3-249D developed a peak titer that was 5-fold lower than the NS3-249P infectious clone (p<0.05), consistent with the smaller observed plaque phenotype at dpi 3. All WN/IC NS3-249 mutants (NS3-249A, NS3-249D, NS3-249P, and NS3-249T) reached peak titers at 48 hours post-inoculation (hpi) with the exception of NS3-249H that peaked 24 hours later (72 hpi) (Fig. 3c).

### Murine neuroinvasive phenotypes of NS3-249 mutants

Three week-old CD-1 mice were challenged by intraperitoneal inoculation with ten-fold serial dilutions of each of the NS3-249 genetic variants (NS3-249T, 249H, 249D and 249A) as well as the parental WN/IC-P991 virus containing the Pro residue at NS3-249. All viruses resulted in morbidity/mortality within 7–8 days post-infection (dpi) and no significant difference (p>0.1) in the calculated lethal dose 50% (LD_50_) was observed between the parental (NS3-249P) and mutant viruses with, LD_50_ values calculated between 0.3 and 0.6 plaque forming units (PFU). LD_50_ values of 0.4 PFU were identified for 249P, 249T and 249A viruses while the 249H and 249D viruses exhibited slightly lower and higher values of 0.3 and 0.6 PFU, respectively.

### Avian viremia/ pathogenesis

**Figure 4:**
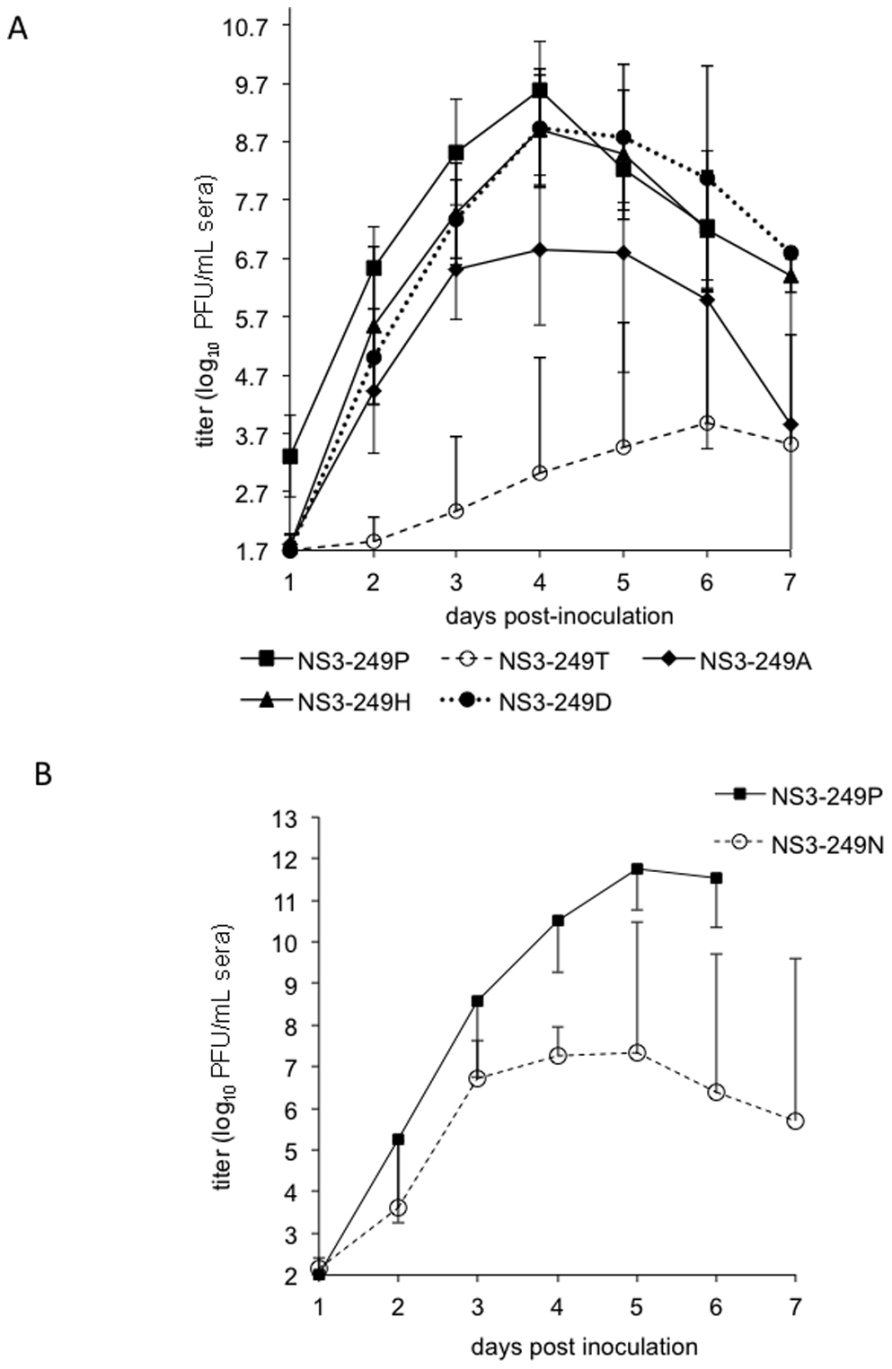
Viremia profiles of AMCRs inoculated with WN/IC NS3-249 mutants. (A) Mean daily viremias from AMCRs (n = 8) from Colorado inoculated with WN/IC NS3-249 point mutants (NS3-249P, 249D, 249T, 249H, and 249A); (B) Mean daily viremias from 2012 captured AMCRs (n = 4) inoculated with WN/IC NS3-249P and 249N mutants. Bars denote standard deviations from the mean.

The expression of various amino acid residues at the WNV NS3-249 position produced a broad range of viremia profiles in the AMCR model (Fig. 4a, 4b). The NS3-P249D and the NS3-P249H substitutions in the NY99 genetic backbone resulted in acute viremia profiles that were indistinguishable in magnitude and duration from the WNV NS3-249P parental strain, except on day 1 (p>0.1) (Fig. 4a). The greatest impact on WNV peripheral virus production occurred with the introduction of the NS3-P249T residue into the WNV NY99 backbone. This amino acid exchange delayed the onset of detectable viremia by 48 hours, drastically reduced the peak viral load from 9.6 log_10_ PFU/mL to 3.9 log_10_ PFU/mL (p<0.0001), and shifted the onset of the mean peak viremia from 4 to 6 dpi (Fig. 4a). The WN/IC NS3-249T mutant was the only virus in which inoculated birds did not develop detectable viral loads (n = 3) during the seven-day experimental study, but these three birds did develop WNV-specific neutralizing antibody titers at fourteen dpi, providing evidence of infection (data not shown). The NS3-P249A mutant exhibited a 49-fold lower mean peak viremia compared to the NS3-249P virus (p = 0.07), whereas AMCRs inoculated with the NS3-249A mutant exhibited 234-fold higher mean titers than those from the attenuated WN/IC NS3-249T virus (p<0.05). In a separate study performed in AMCRs collected in 2012, viremia profiles from AMCRs inoculated with the NS3-249N mutant were compared directly to those inoculated with the wild type Pro virus. Similar to the differences observed with the NS3-249A mutant, the NS3-249N virus elicited a peak viremia at dpi 5 that was 10,000-fold lower (p<0.01) than that observed with the NS3-249P virus (Fig 4b).

**Figure 5:**
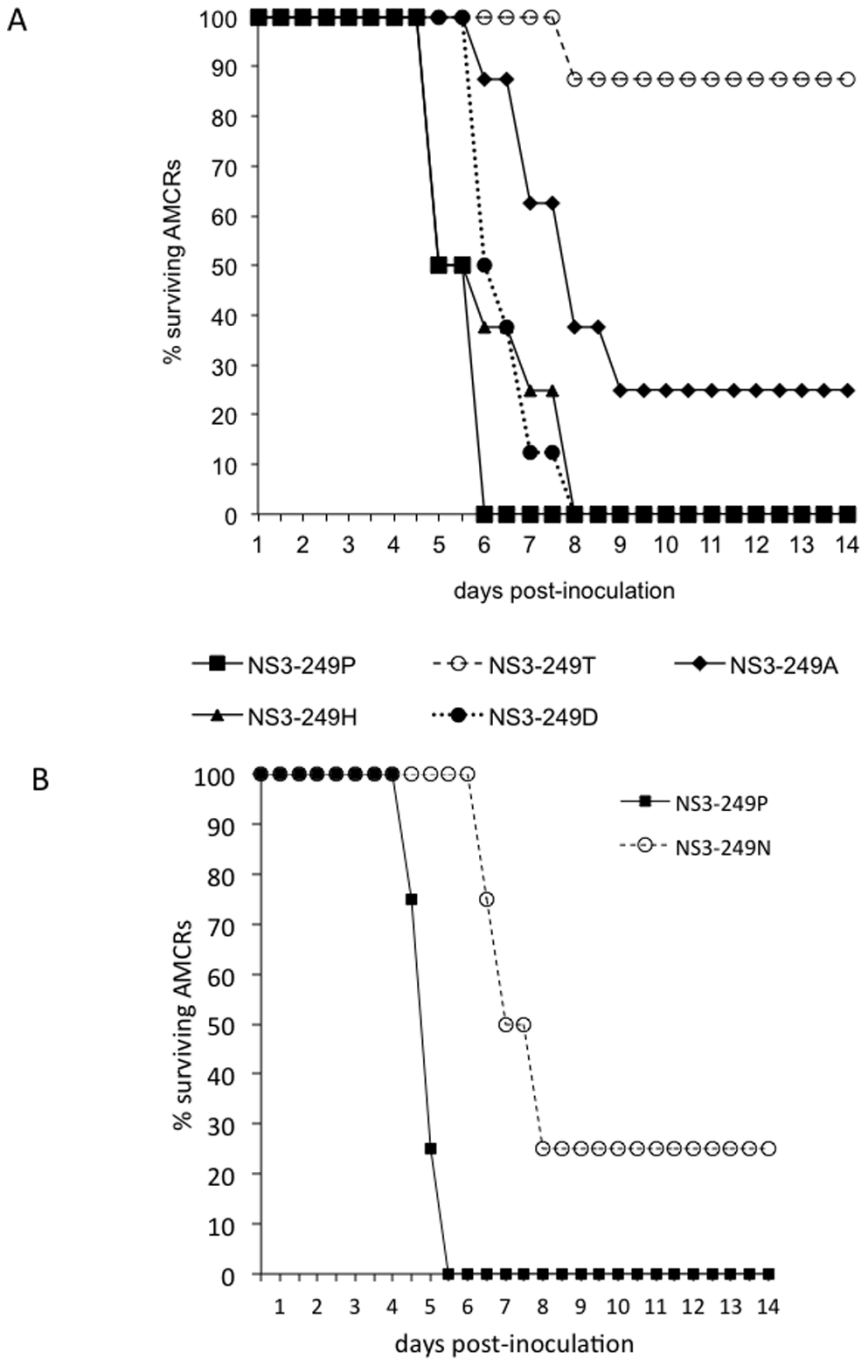
Survivorship of AMCRs inoculated with WN/IC NS3-249 mutants. (A) Survivorship of Colorado AMCRs (n = 8) inoculated with WN/IC clone-derived viruses demonstrating variable amino acids at the NS3-249 locus (249P, 249D, 249T, 249H, and 249A); (B) Survivorship of AMCRs collected in 2012 (n = 4) inoculated with WN/IC clone-derived viruses (NS3-249P and 249N).

Because the WN/IC NY99 prototype virus induces high peripheral viral loads and 100% mortality when inoculated in AMCRs, the importance of introducing alternative NS3-249 amino acid substitutions on avian survivorship was assessed (Fig. 5a, 5b). The impact of alternative WNV NS3-249 residues on the survivorship of AMCRs was dependent on the particular amino acid introduced into the NY99 genetic backbone. The NS3-P249T substitution constituted the AMCR group with the highest percent survivorship (87%) [19] followed by the NS3-P249A construct (25%). The high sustained viral loads observed in AMCRs inoculated with the NS3-P249D and NS3-P249H constructs resulted in no survivorship, but slightly delayed the mean onset of death by 2 days when compared to the WNV NS3-249P. AMCRs collected in 2012 inoculated with the wild type NS3-249P virus also resulted in 100% mortality within six days of inoculation, while the NS3-249N mutant demonstrated only 25% mortality, concomitant with 10,000-fold lower viremia (Fig. 5b).

**Figure 6:**
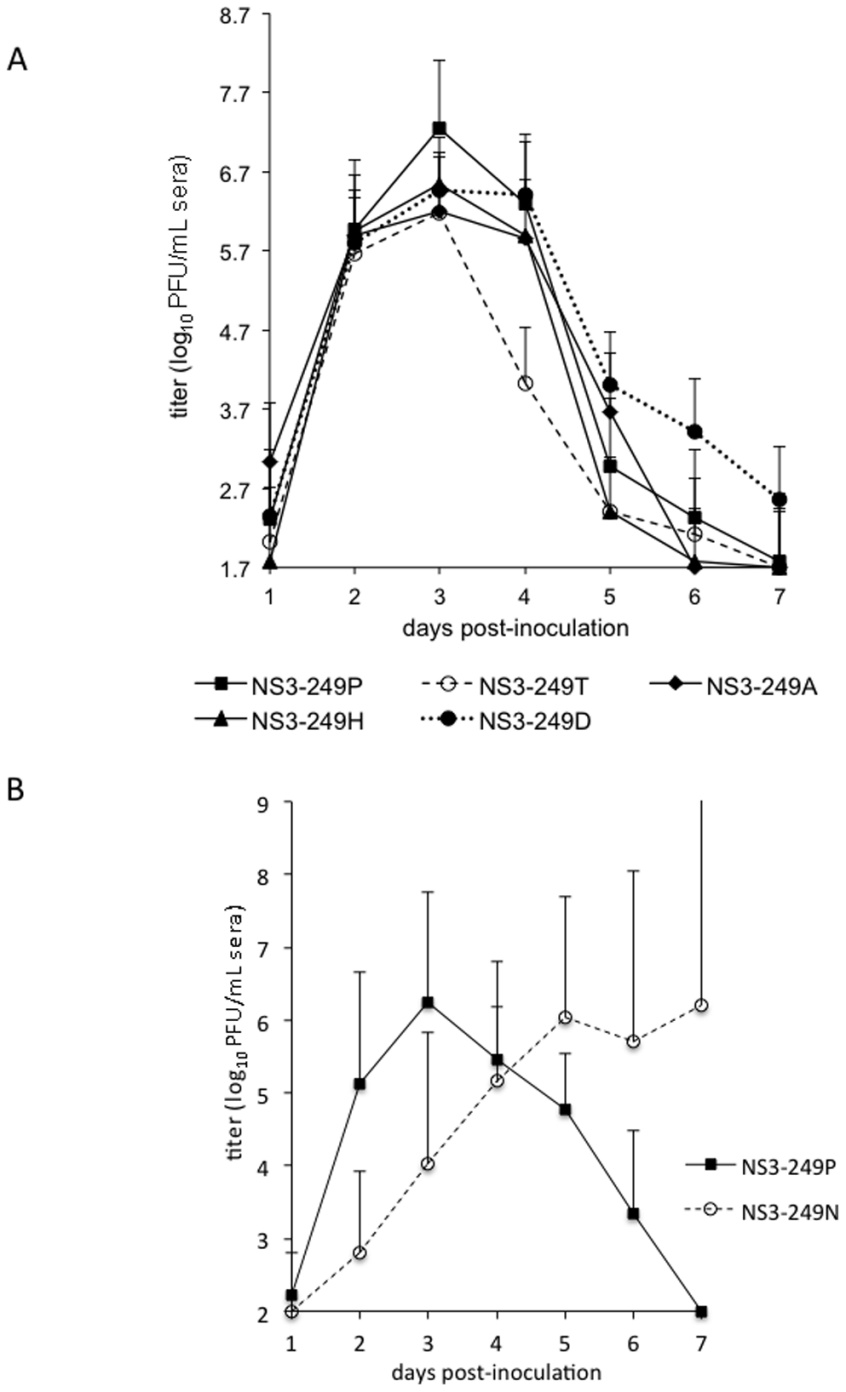
Viremia profiles of HOSPs inoculated with WN/IC NS3-249 mutants. (A) Mean daily viremias from HOSPs (n = 8) from California inoculated with WN/IC NS3-249 point mutants (NS3-249P, 249D, 249T, 249H, and 249A); (B) Mean daily viremias from HOSPs (n = 4) from Colorado inoculated with WN/IC NS3-249P and 249N mutants. Bars denote standard deviations from the mean.

Modification of the WN/IC NS3-249 Pro residue to an Ala, Asp or Thr had little effect on HOSP viremia profiles (Fig. 6a). The WN/IC NS3-249 variants (NS3-249A, NS3-249D, NS3-249H, and NS3-249T) did not significantly impact the mean peak viral loads in the HOSP model compared to the NS3-249P (p>0.5). All virus titers for Pro (wt) and Thr, Asp, His and Ala mutants peaked in the peripheral blood on 3 dpi in the HOSP model; however, the NS3-249T mutant developed mean peak viral titers approximately 10-fold lower than the NS3-249P on day 3 dpi. In contrast, the NS3-249H substitution in the WN/IC backbone reduced the mean peak viral load by 200-fold, leading to viral titers below the threshold to infect most *Culex* spp. mosquito species. In a separate experiment, HOSPs collected in 2012 were inoculated with the wild type Pro and Asn mutant viruses. Peak viremias of approximately 6 log_10_ PFU/mL sera were observed in both infection groups; however, the peak titer was delayed from dpi 3 (Pro) to 5 dpi or later for the Asn mutant (Fig. 6b).

### WNV complete genome and NS3 subgenomic sequence analysis

In order to observe avian host selection pressures potentially exerted on WNV populations containing different amino acid residues at the NS3-249 site, viral RNA was extracted from AMCR peripheral blood at the WNV peak viral load for each bird and NS3 RT-PCR amplicons were generated for sequencing. Crow sera collected at peak viral loads, determined by Vero cell plaque assay, were subjected to viral RNA extraction and WNV NS3 helicase RT-PCR amplicons were sequenced to assess reversions within the helicase gene, particularly at the NS3-249 residue. The NS3-249D and NS3-249H viruses isolated from AMCR sera at peak viral titers on 4-6 dpi did not contain any amino acid substitutions within the helicase region sequenced. All crows inoculated with WNV NS3-249A virus retained the alanine amino acid residue at NS3-249 with the exception of one bird that was found to have a viral genetic polymorphism at the NS3-249 position on 5 dpi. Consensus sequence analysis of the amplicon generated from AMCR 1, the bird with one of the highest viremias at 9.4 log_10_ PFU/mL, demonstrated a minority subpopulation CCC codon at the amino acid codon encoding a Pro residue. This attenuated WN/IC mutant (NS3-249A) also picked up a non-synonymous substitution (Asp to Glu) ten amino acids downstream at the NS3-259 residue in two infected AMCRs, AMCR-2 and AMCR-6. Two AMCRs, AMCR-1 and AMCR-7, inoculated with the highly attenuated WN/IC NS3-249T had viral populations with an amino acid substitution, Gln to His, at the NS3-244 residue.

### Temperature Sensitivity of NS3-249 mutants

**Figure 7:**
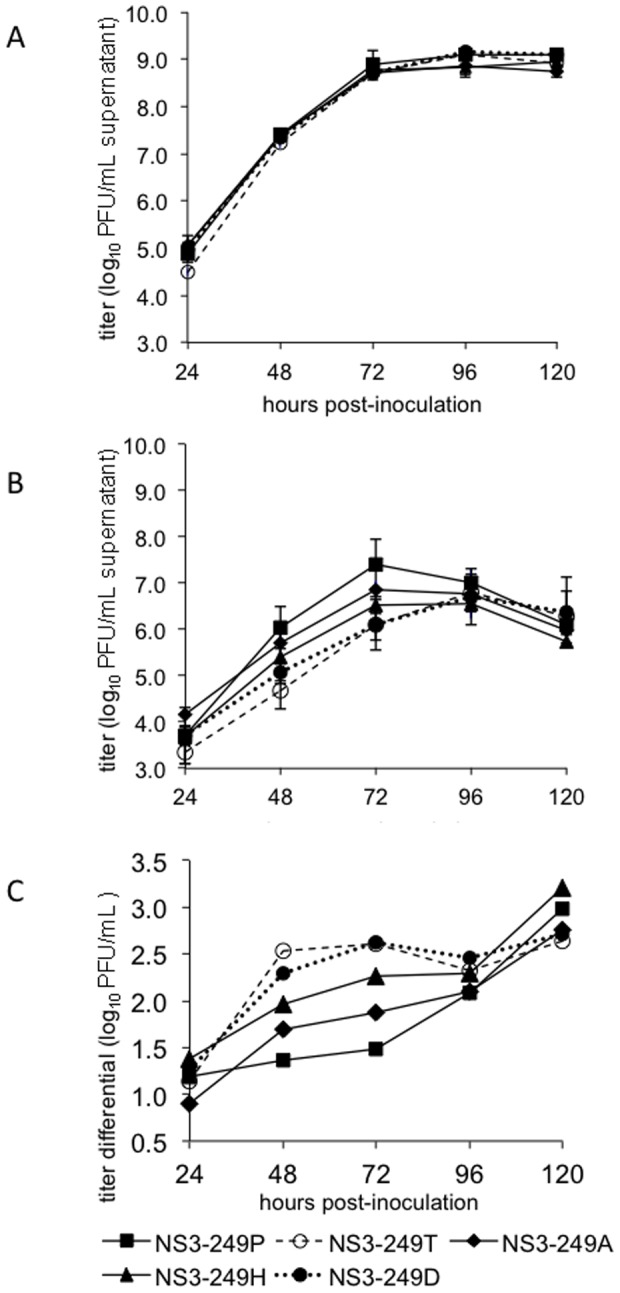
Temperature sensitivity assessment in an avian (DEF) cell line performed at 37°C (A) and 44°C (B). Cells were inoculated at an MOI of 0.1 and titers determined by plaque titration on Vero cells. (C) Depicts a compilation of the differential growth by subtracting titers determined from growth at 37°C from those observed at 44°C.

In order to assess whether the differential growth phenotypes of the NS3-249 polymorphisms could be related to differential capacity of the viral mutants to replicate at the higher physiological temperatures observed in WNV-infected AMCRs, NS3-249 mutants were grown in duck embryo fibroblast cells (DEF) at highly permissive (37°C; Fig. 7a) and elevated temperatures observed in febrile AMCRs (44°C; Fig. 7b) [26]. No significant differences were observed in virus production of any of the NS3-249 mutants at 37°C, with all viruses generating ≥8 log_10_ PFU/mL of culture supernatant by 72–96 hpi (Fig. 7a). In contrast, a range of temperature sensitivities were observed at 44°C (Fig. 7b), with the Pro virus generating the highest mean titer of 7.4 log_10_ PFU/mL supernatant at dpi 3 and the Thr mutant demonstrating the most consistently temperature sensitive phenotype, peaking at 6.8 log_10_ PFU/mL at dpi 4 (Fig. 7b). A direct comparison of differences in the growth potential of the viral mutants at the two temperatures demonstrated a clear range of temperature sensitivity phenotypes (Fig. 7c). At 72 hpi, the Pro virus demonstrated only a 20-fold reduction in titer at 44°C compared to 37°C, while the Ala showed a 50-fold decrease and the Thr mutant exhibited a 300-fold reduction in titer (Fig. 7c). These differences accurately modeled the *in vivo* replication phenotypes observed in AMCRs; nevertheless, the Asp mutant was as temperature sensitive as the Thr mutant, yet demonstrated a high AMCR replication phenotype. The NS3-249H virus exhibited an intermediate temperature sensitive phenotype and also replicated well in AMCRs.

### Helicase activity of WNV NS3-249 point mutations

**Figure 8:**
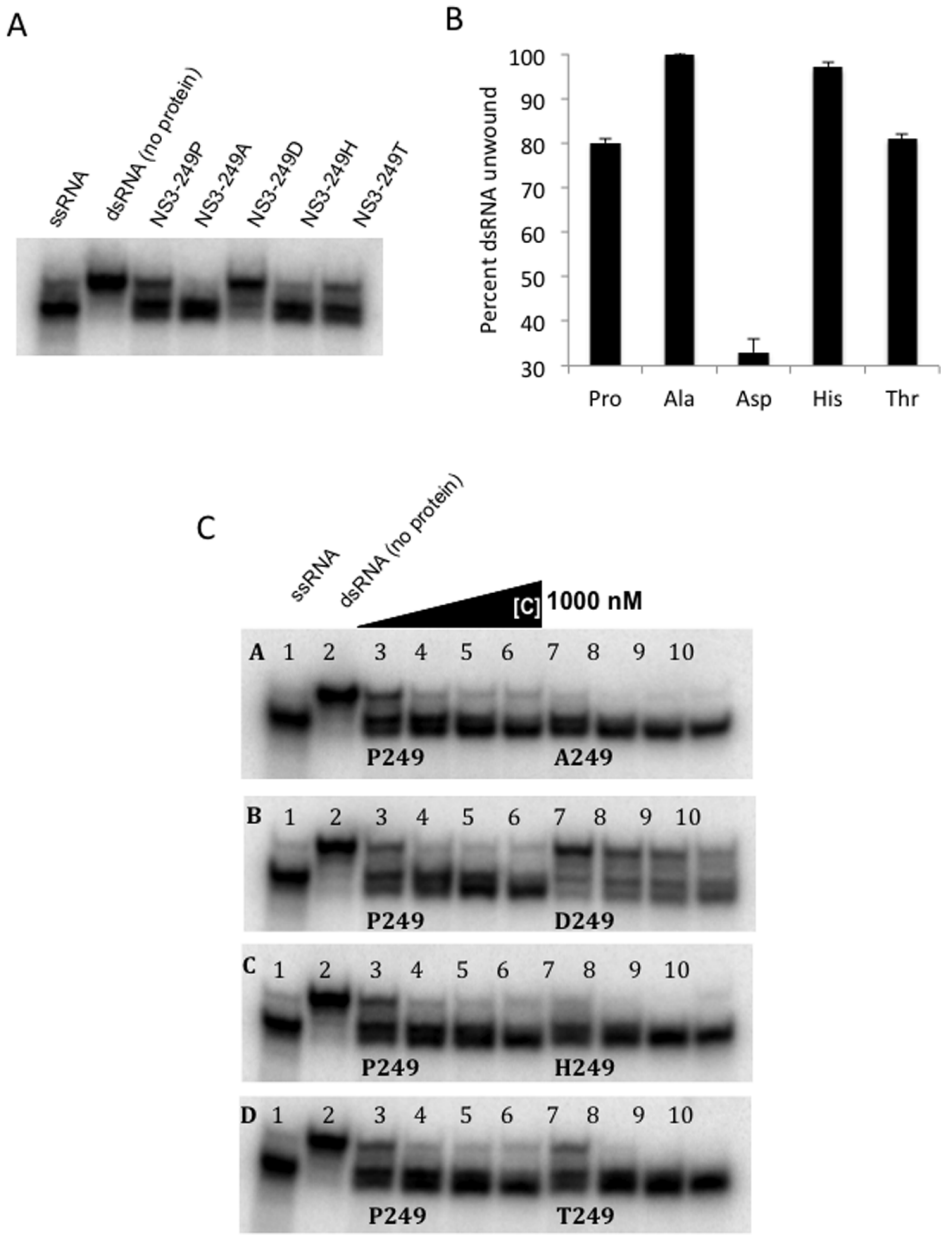
Helicase activity of recombinant WNV helicase NS3-249 protein mutants. (A) Helicase activity with fixed concentration of NS3 (125 nM). Lane 1: ssRNA (heat denature dsRNA), Lane 2: dsRNA (no protein), Lane 3: NS3-249P, Lane 4: NS3-249A, Lane 5: NS3-249D, Lane 6: NS3-249H, Lane 7: NS3-249T. (B) Percent dsRNA unwound by each NS3-249 helicase point mutants at 125 nM. (C) Helicase activity with increasing concentrations of NS3 (125 nM, 250 nM, 500 nM and 1,000 nM).

In order to determine the impact of non-conservative amino acid substitutions at the NS3-249 residue on helicase function, recombinant helicase protein containing NS3-249 point mutations were assessed for their respective capacities to unwind short double-stranded (ds) RNA (Fig. 8). At 125 nM of each WNV NS3 protein, the helicase activity greatly varied when comparing NS3 proteins containing alternative NS3-249 point mutations. The NS3-249 proteins containing the alanine and histidine residues unwound >95% of the dsRNA molecules, whereas the NS3-249 proteins with the proline and threonine point mutations separated >80% of the dsRNA strands. In contrast, the NS3-249D protein only unwound ∼33% of the dsRNA molecules showing a debilitation in helicase activity when compared to the other NS3-249 proteins. Although the helicase differed between the variants, these differences did not correlate with the *in vivo* effects observed in AMCRs.

### ATPase activity of WNV NS3-249 point mutants

**Figure 9:**
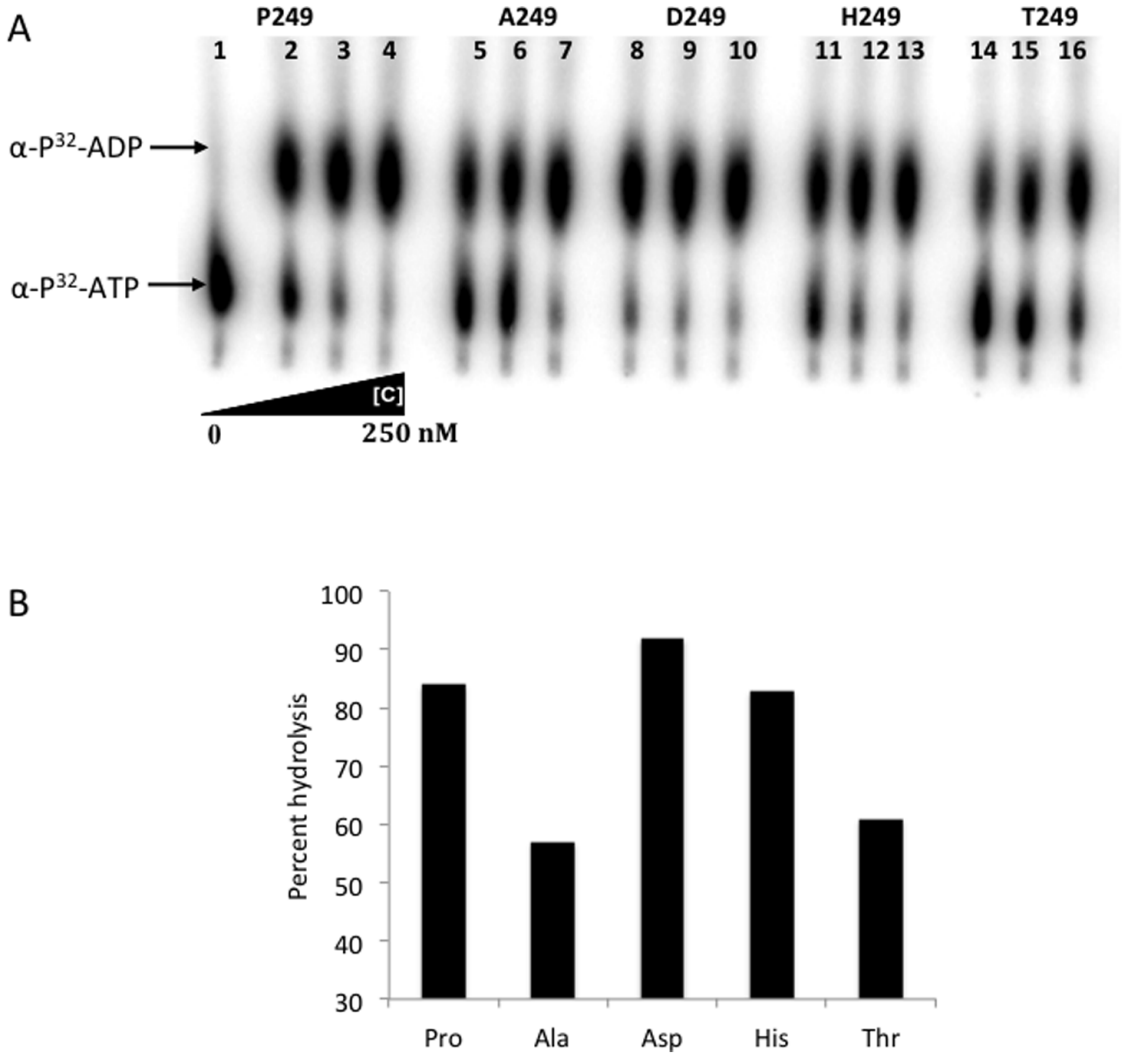
NS3-249 point mutant ATPase kinetics during dsRNA unwinding. (A) ATPase activity with different concentration of NS3 protein. Lane 1: no protein, with 62.5 nM of recombinant protein (lane 2, 5, 8, 11 and 14), 125 nM of protein (3, 6, 9, 12 and 15), and 250 nM of protein (lane 4, 7, 10, 13 and 16). (B) Percent ATP hydrolyzed by each NS3-249 helicase protein at concentrations of 125 nM.

Because the WNV NS3-249 residue resides in close proximity to the viral ATP binding domain, the efficiency of alternative recombinant NS3-249 helicase proteins to hydrolyze ATP molecules was assessed (Fig. 9). All WNV NS3-249 helicase proteins demonstrated the capacity to hydrolyze ATP in a dose-dependent manner. At 125 nM of each NS3 helicase protein, the NS3-249 point mutant proteins, NS3-249D, NS3-249H, and NS3-249P, maintained the greatest ATPase activity, hydrolyzing >80% of ATP to ADP. In contrast, the NS3-249A and NS3-249T helicase proteins exhibited reduced ATPase activity (<60%).

## Discussion

The recent expansion of WNV throughout the Western hemisphere and its re-emergence worldwide has highlighted questions concerning the introduction of specific WNV genotypic mutations that have enhanced avian transmission capacity leading to larger WNV epizootics. Naïve avian populations, moderately competent vector mosquito species, and WNV phenotypic adaptations to augment vector [28] and host competence [19] are possible driving forces that led to the expeditious spread of WNV across the continental U.S. in less than 5 years. Previous studies have identified a critical genetic determinant within the helicase domain of the NS3 protein (NS3-249) that modulates WNV pathogenesis in the AMCRs [19], [25]. Introduction of an NS3-P249T substitution in a North American East Coast lineage 1a genotype backbone (NY99) restricted peripheral viral replication and dramatically increased the survivorship in AMCRs [19]. Selection modeling coupled with the phenotypic evidence that this site modulates avian replication provided further support that replicative capacity in an avian host could be an instrumental feature for the emergence of different WNV lineages. A comprehensive study involving sequence alignments of 146 WNV NS3 proteins demonstrated this protein to be highly conserved among WNVs with a maximum divergence of 10% at the amino acid level. Highly conserved sequence motifs within the NS3 helicase flank the NS3-249 position (NS3-235-243 and NS3-256-282) and the NS3-256-282 motif comprises a portion of the DEAD/H binding domain involved in phosphorylation of protein kinase C (Fig. 2a) [29]. Interestingly, amino acid alignments of WNV demonstrate that a series of hydrophobic residues (NS3-243-254) within the NS3 helicase that are highly conserved among WNVs, with variation being observed only at the NS3-249 residue (Fig. 2a). The NS3-249 residue is positioned on the terminus of the hydrophobic loop and would be in a strategic orientation for direct interaction with alternative viral or host proteins (Fig. 2b). Furthermore, this positioning would allow for any number of amino acids to be present at this loci and, as such, explains the potential for the variety of lineage-specific amino acid identities observed at this site.

The introduction of an NS3-threonine at position 249 in the NY99 backbone was sufficient to significantly lower viral loads and delay the detection of circulating infectious virus by 48 hpi in the AMCR model. The delay in the production of peripheral WNV virus due to differential replication kinetics in early target host cells could allow for the AMCR immune response to sufficiently control viral spread through the activation of the innate and adaptive immune responses [30]. This could explain the seropositive WNV NS3-249T AMCRs that did not develop a detectable viremia, but still stimulated a WNV-specific humoral immune response at 14 dpi. As observed in the HOSP model inoculated with the WNV KN98 (NS3-249T) strain containing a threonine at the NS3-249 residue, the NS3-249T mutant did not produce significantly lower mean peak viral loads when compared to the NS3-249P virus [31]. In contrast to the AMCR model, the WNV NS3-249T virus produced high titers, detectable in all inoculated HOSPs. High titers were observed in all HOSPs during the first 48 hrs of acute infection but exhibited a rapid decline following peak viremia on dpi 3. The lack of sustained infectious viral loads (>5 log_10_ PFU/mL blood) in both avian models when compared to the WNV NS3-249P could be one potential mechanism to explain why WNV NS3-249T genotypes have not been associated with large epizootics in endemic regions.

Mutant viruses with alternative amino acids at the NS3-249 residue that demonstrated an attenuated phenotype, NS3-249A and NS3-249T, in the AMCR model incorporated additional amino acid substitutions within domain I of the NS3 helicase in a subset of birds. Both WN/IC NS3-249T mutants had amino acid substitutions at the NS3-244 loci (NS3-Q244H). The WNV NS3-Q244H (Fig. 2a and 2b) substitution has been documented in another WNV isolate from France (2000)(Fig. 2a) that contains the threonine residue at the NS3-249 position [32], [33]. The introduction of a histidine could assist in stabilizing the NS3 hydrophobic loop moiety when a threonine is present at the NS3-249 residue, but further studies are needed to characterize the role of stabilization of the hydrophobic domain as a function of avian peripheral replication. When the NS3-249A residue was expressed within the aforementioned domain (NS3-243-254)(Fig. 2a and 2b), non-synonymous mutations were identified at the NS3-D259Q residue in two of eight viremic AMCRs. The NS3-259D (Fig. 2a and 2b) residue is part of the DEAD/H binding domain and is highly conserved in all WNV NS3 proteins analyzed to date [29]. The location of this WNV NS3 motif within viral RNA and host protein kinase C phosphorylation binding domains coupled with the identification of additional non-synonymous mutations in the attenuated WNV NS3-249 mutants (NS3-249A, NS3-249T) during avian replication suggests this surface protein moiety interacts with a critical host protein during acute infection in the AMCR model.

The evaluation of helicase activity on the unwinding of short dsRNA by alternative WNV NS3-249 helicase proteins did show differences between the amino acid substitutions (249A, 249D, 249H, 249P, 249T), but the *in vitro* results did not correlate well with the *in vivo* avian virulence studies. Further helicase analysis under more relevant experimental conditions, i.e. higher more biologically relevant temperatures [26], will be necessary to fully address this hypothesis. The effect of WNV NS3-249 amino acid substitutions on ATPase activity appeared to be functionally coupled with the virulence potential observed in the AMCR model, but not the HOSP model. These data suggest that the NS3-249 residue could be important for viral helicase ATP hydrolysis in one host species, but have minimal functionality in another host species potentially due to direct interaction between the NS3-249 residue and unknown host factors. Interestingly, the changes in helicase activity were inversely correlated with changes in ATPase activity. The NS3-249A mutant demonstrated the highest levels of ATPase and the lowest helicase activity whereas the NS3-249D mutant exhibited the most active ATPase and the weakest helicase activities (Figs. 8 and 9).

Growth phenotype differences between AMCRs and HOSPs were unanticipated because both birds are in the order *Passeriformes*, although HOSPs were introduced intentionally into the northeastern USA from Europe ca. 160 years ago [34]. Growth of the viral mutants at elevated temperatures in DEF cells did demonstrate superior replication of the NS3-249P virus compared to the other mutants with the NS3-249T mutant demonstrating the greatest level of temperature sensitivity; however, there was discordance between AMCR growth phenotypes and temperature growth phenotypes for a number of the mutants. Similar discordances were observed in HOSP growth phenotypes with various NS3-249 mutants. This finding, coupled with the observation (unpublished data) that HOSPs develop hypothermia (unlike AMCRs) [26] following WNV infection indicate the potential that growth at elevated temperature could be a contributing factor to the observed AMCR phenotypes of the mutants assessed.

The reverting and/or compensatory mutations introduced into the WNV NS3 helicase, within the hydrophobic motif encompassing the NS3-249 residue, in NS3-249A and NS3-249T mutants that demonstrated an attenuating phenotype in the AMCR model suggests this loop motif is critical for development of high levels of peripheral infectious virus. The NS3-249 helicase mutants (NS3-249D, NS3-249H) that were highly virulent and replicated to high viral titers in the AMCR model appeared to be stable in the NY99 genetic backbone. Most of the NS3-249 point mutants replicated well in both AMCRs and HOSPs; however, the finding that the NS3-249T mutant was highly attenuated in AMCRs but replicated well in HOSPs suggests that host protein interactions with this motif could result in species-specific replication differences. Furthermore, it is possible that alternative NS3-249 amino acids could modulate infection, dissemination and/or transmission in enzootic mosquito vectors or facilitate replication in alternative avian hosts important for enzootic maintenance. In addition, the role of alternative genetic changes in different lineage 1 and alternative lineages that have polymorphisms at this locus could have significant effects on fitness in different hosts. NS3-249 polymorphisms were observed to have emerged on at least twelve different occasions (Fig. 1b and 2a). Notably, eight of these were NS3-T249P emergence events in lineage 1a backbone viruses (with one lineage 2 NS3-H249P event in 2010), further supporting the contention that the genetic backbone could modulate fitness expression and potentially restrict the incorporation of genetic changes at NS3-249 for all WNV lineages.

Previous studies identifying the evolutionary pressures on this particular NS3-249 residue could partially explain the epizootic potential of WNV strains that have incorporated a proline at this position in the genome. Elevated infectious titers and the longer duration of sustained infectious viremias across an extensive range of bird species would allow for higher infection rates in susceptible mosquito species and increase the potential for infection of bridge vectors that primarily feed on mammals. Furthermore, the fact that different amino acid residues present at the WNV NS3-249 site had no effect on mammalian virulence indicates that this observed phenomenon is likely avian specific. Together, these results could help to explain the rapid dissemination of North American WNV strains throughout the Western Hemisphere and Europe and the increase in WNV human/equine cases reported in the last decade.

## Materials and Methods

### Sequence Analyses of West Nile viruses

An alignment of the coding region of 36 WNV strains (genomic positions 97-10,399) was made using Clustal Omega [35]. jModelTest2 was used to determine the best-fit nucleotide substitution model for the data [36], [37]. Based on these results, a GTR + I + Γ model was used in further analyses. A Bayesian phylogeny was constructed using MrBayes [38], accessed through Topaliv2.5 [39], with multiple runs of 1,000,000 generations and 50% burn-in. Maximum Likelihood analysis was performed using PhyML [40] with 1,000 bootstraps. Bayesian tests for selection acting on individual sites were performed using FUBAR in the HyPhy package accessed through Datamonkey [41]. Genbank accession numbers of sequences used: Koutango virus EU082200; Australia 1960 Kunjin virus D00246; Australia 2011 JN887352; Morocco 1996 AY701412; Czech Republic 1997 Rabensburg strain AY765264; Hungary 2003 DQ118127; Russia 2007 FJ425721; Sarafend strain AY688948; South Africa 1989 EF429197; South Africa 2001 EF429198; Uganda 1937 M12294; Egypt 1951 AF260968; Greece 2010 HQ537483; India 1968 EU249803; Israel 1998 AF481864; Italy 2008 FJ483549; Mexico 2003 AY660002; New York 1999 AF196835; Portugal 2004 AJ965628; Romania 1996 AF260969; Russia 1999 AF317203; Russia 2001 DQ411029; Spain 2007 FJ766331; China 2001 AY490240; Ethiopia 1976 AY603654; France 2000 AY268132; India 1980 DQ256376; Italy 1998 AF404757; Italy 2008 JF719065; Kenya 1998 AY262283; Morocco 2003 AY701413; Russia 1998 AY277251; Spain 2008 JF707789; Spain 2010 JF719069; Tunisia 1997 AY268133; Hungary 2004 DQ116961.

### WNV infectious cDNAs and plasmids

Generation of infectious WNV NY99 constructs have been described previously [42]. The non-synonymous amino acid point mutations (Ala, Asp, His, Thr and Asn) were introduced into the WN/IC NY99 backbone (CG plasmid) using site-directed mutagenesis (Table 1) as previously described [43]. Following *in vitro* ligation of the AB (5′) and CG (3′) plasmids, XbaI linearization at the 3′ terminus of the WNV genomic cDNA was performed to serve as a template for run-off transcription. The full-length genomic WNV DNA was treated with proteinase K (New England Biolabs), extracted with phenol: chloroform: isoamyl alcohol, and then chloroform, and precipitated with ethanol and sodium acetate. The DNA pellets were resuspended in 20 µl of RNase-free water and viral genomic RNA was transcribed for 2 hours at 37°C using the AmpliScribe T7 transcription kit (Epicentre). Reactions included 6 mM m^7^G-(5′)ppp-(5′)A cap analog (New England Biolabs), 20% of the manufacturer-recommended concentration of ATP, and 0.5–2 µg of *in vitro*-ligated pWN-AB/pWN-CG DNA, and the newly transcribed RNA was transfected by electroporation into BHK-21 cells using a BTW petri-pulser (Biorad) [42]. Transfected cells were incubated at room temperature for 15 min, transfection culture was removed, and 4.5 mL of MEM containing 5% pen-strep with 10% FBS was added. Culture medium was harvested when cytopathic effects (CPE) were clearly evident (∼60%), clarified by centrifugation to remove cellular debris and distributed in aliquots for storage at −80°C. Virus titers were determined by plaque assay [25].

### Genomic and partial genomic sequencing

RNA was extracted from rescued viruses, cDNA was generated and sequencing [44] of the complete genomes of all viruses was performed to confirm the genetic identity of all viruses utilized for avian virulence testing. Sequencing of WNV cDNA within plasmids, and overlapping cDNA fragments amplified from viral RNA genomes by RT-PCR, was performed using previously described protocols [45]. The extreme 5′-terminal sequence of each WN/IC NS3-249 mutant RNA genome was determined by using the 5′RACE (Invitrogen) method [42]. Similarly, the extreme 3′-terminal sequence was determined by employing *E. coli* poly(A) polymerase to tail the RNA with poly(A), followed by RT-PCR using virus-specific and oligo(dT) primers, as described previously [46]. Total RNA was extracted directly from AMCR sera at 4 or 5 dpi using Trizol LS (Invitrogen) or a viral RNA extraction kit (Qiagen) to avoid confounding cell culture passage-related sequence changes. RNA was reverse transcribed and PCR amplified to generate a 900 nt amplicon using a 1-step RT-PCR kit (Invitrogen). The primers used to reverse transcribe the cDNA template and DNA amplicons were: forward primer- 5′-CAGGGTGAAAGGATGGATGAG-3′ and reverse primer- 5′-CACCAACTTGCGACGGATTTG-3′. All WNV amplicons were sequenced using a capillary sequencer.

### Murine virulence assessment

Groups of five 3 week-old CD-1 mice (Charles River) were inoculated by intraperitoneal injection of 0.1 mL PBS suspension of serial 10-fold dilutions of each of the WNV parental (NS3-249P) and four WNV mutants (NS3-249T, 249A, 249H and 249D) from 0.1 PFU to 1,000 PFU as previously described [47]. Mice were monitored daily for signs of disease (ruffled fur, hunching or limb paralysis). Mice demonstrating limb paralysis or restricted movement in addition to ruffled fur were euthanized. Lethal dose 50% endpoints (LD_50_) were calculated using the Spearman-Karber method.

### *In vitro* growth profiles

Diameters of at least 6 well-defined plaques were measured on Vero cells in 6-well plates from monolayers inoculated with each virus (Pro, Thr, Ala, His, Asp and Ala mutants). Inoculated monolayers were overlaid with 0.4% agarose plaque diameters measured at dpi 3. Briefly, digital images of wells were taken at dpi 3 and plaque diameters calculated from the reference diameter of the well. Duck embryonic fibroblast cells (DEF; ATCC, CCL-141) and African green monkey kidney cells (Vero; ATCC, CCL-81) were utilized to assess replicative fitness of the WN/IC NS3-249 point mutants in avian and mammalian cells *in vitro*. All viruses (n = 5) were inoculated at an MOI ∼ 0.1 onto monolayers of Vero and DEF cells in triplicate using a 6-well plate format (Vero) or 25 cm^2^ flask (DEF). WN/IC point mutants were allowed to adsorb for 1hr at 37°C, inoculum was removed, cells were washed 3X with PBS, and 4.5 mL of DMEM (Vero) or 6 mL Eagle's MEM (DEF) (Invitrogen) with 5% (Vero) or 10% (DEF) FBS added to each well/flask, respectively. For temperature sensitivity studies in DEF cells, replicate experiments were performed at both 37°C (control temperature) and 44°C (high temperature). Supernatant from inoculated plates was removed (700 µL) from individual replicate wells every 24hrs for 5 days and mixed 1∶1 with DMEM containing 20% FBS. 50 µL of supernatant was removed from triplicate DEF flasks at 24hr intervals, diluted 1∶10 in EMEM with 20% FBS and frozen at −80°C until assayed for viral titer. Infectious viral titers in the supernatants were quantified using standard plaque assay techniques on Vero cells.

### Avian experimental studies

After hatch-year AMCRs were netted in 2005 under US Fish and Wildlife Services and Colorado Parks and Wildlife permits with permission of Morning Fresh Dairy (40° 38’ 51”, 105° 11’ 15” W) as well as the managers of the Colorado State Fisheries Unit (40° 37’ 35” N, 105° 10’ 32” W) in Bellvue, CO, banded and bled to determine pre-existing neutralizing antibodies against WNV and St. Louis encephalitis viruses (SLEV) and housed at the Colorado State University Animal Disease Laboratory in groups of two within 1-m^3^ cages. Crows were fed an *ad libitum* mixture of dry cat and dog food as previously described [25]. In order to control for dose delivered as well as to control for the genetic identity of administered recombinant viruses, viruses were administered by subcutaneous inoculation rather than mosquito bite. One hundred microliters (100 µL) of the diluted primary transfection culture containing 1,500 PFU of the parental (NS3-249P) and NS3-249T, 249A, 249H and 249D mutants were administered subcutaneously by needle on the breast region of eight AMCRs per virus. To control for temporal variation in susceptibility of AMCRs to WNV, the parental NS3-249P virus was also used for inoculation of an additional group of four AMCRs collected in Colorado in 2011 along with an infection group with the lineage 3 Asn mutant (n = 4). HOSPs were collected in Bakersfield, CA and Fort Collins, CO using Japanese mist nets and box-baited traps. All captured birds were banded, tested for pre-existing infections to SLEV and WNV by PRNT, and housed in a mosquito-proof aviary prior to *in vivo* experimentation. Groups of six HOSPs from Bakersfield were inoculated with 1,500 PFU of the parental NS3-249P clone-derived WNV and each NS3-249 point mutant (Ala, His, Asp and Thr) on the breast/cervical region by 28 gauge needle. Similarly, HOSPs from Ft. Collins, CO were inoculated with the Asn mutant as well as the NS3-249P in order to control for variations in geographic susceptibility. All HOSPs were checked twice daily for clinical signs of infection, and fed *ad libitum* with a multi-seed mixture supplemented with mealworms. AMCRs and HOSPs from each experimental group were bled once daily by either jugular or brachial venipuncture with a 26-gauge needled syringe (AMCRs) and 28-gauge needled syringe (HOSPs) from 1–7 dpi and monitored daily for signs of disease through 14 dpi. Each 0.2 mL blood sample (AMCRs) was added to 0.8 mL of BA-1 diluent (Hanks M-199 salts, 0.05 M Tris pH 7.6, 1% BSA, 0.35 g/L sodium bicarbonate, 100 U/mL penicillin, 100 µg/mL streptomycin, 1 µg/mL Fungizone). HOSPs were also bled daily through 7 dpi by jugular venipuncture, but 0.1 mL of whole blood was added to 0.45 mL of diluent. Coagulation was allowed to take place at room temperature for 30 min. The samples then were placed on ice, centrifuged for 10 min at 4000-x g, and frozen at −80°C until titrated for infectious units using standard plaque assay techniques. Birds were checked at least twice daily and any birds displaying ataxia, incoordination or trouble feeding were euthanized by intravenous phenobarbital overdose. All surviving birds were similarly euthanized at dpi 14 in the same manner.

### Viral Titrations

Infectious virus was assayed by plaque titration on Vero monolayers using 6-well plates as described previously [25]. Plaque forming units were read at 3-4 dpi by adding a second overlay containing 0.05% neutral red on the second dpi. Limits of detection were calculated as 1.7 log_10_ PFU/mL for serum samples and 100 PFU/mL for cell culture medium. Inocula for all viruses were back titrated to confirm the uniformity of doses administered to AMCRs and HOSPs and to approximate the multiplicity of infection on Vero cells.

### Construction of recombinant WNV NS3 helicase plasmids

The WNV NS3 helicase genes containing the wild type (Pro) and 4 individual NS3-249 point mutations (Ala, Asp, His, Thr) were amplified using PCR specific primers that contained BamHI (WN.Hel1- 5′TCGAACCTCATATGCTGAGGAAAAAACAGATCACT3′) and NdeI (WN.Hel2-5′TTTCTTGGATCCTTAACGTTTTCCCGAGGCGAAGTC3′) restriction sites incorporated into the primer sequences. All PCR products were sequentially digested using BamHI and NdeI restriction enzymes and ligated into a pET-28 protein expression plasmid using T4 DNA ligase (New England Biolabs) by incubating for 1hr at room temperature. Ligated plasmids were electroporated into XL-1 blue electrocompetent cells (Stratagene), individual colonies were selected and propagated overnight at 37°C in a bacterial shaker, and the entire cloned viral NS3 domains were sequenced using M13 forward and reverse primers.

### Expression and purification of recombinant NS3 helicase proteins

The N-terminal, His_6_-tagged, recombinant WNV NS3-249 point mutations were purified as described previously [48], [49]. Cultures of *E. coli* strain Rosetta2 (DE3) (Novagen) transformed with the expression plasmid were grown in 1 L of LB medium containing 35 µg/mL chloramphenicol and 50 µg/mL kanamycin at 37°C until OD_600_  =  0.5. The temperature was reduced to 24°C, expression of the recombinant protein was induced by addition of IPTG to a final concentration of 0.4 mM, cultures were incubated for an additional 12 h at 24°C, and cells were harvested by centrifugation. Cell pellets were resuspended in 30 mL lysis buffer (25 mM Na-phosphate pH 6.5, 300 mM NaCl, 20 mM imidazole, 5% glycerol), lysed by three passes through a French Press at a pressure of 1000 MPa, and centrifuged at 27,000 x *g* for 30 min at 4°C. The supernatant was loaded onto a 5-mL HiTrap chelating column (GE Healthcare) pre-equilibrated with lysis buffer. The column was washed with 30 mL Buffer A (25 mM Tris-HCl pH 8.0, 300 mM NaCl, 5% glycerol) containing 50 mM imidazole. The protein was eluted with a linear gradient of 50–300 mM imidazole in Buffer A. Fractions containing NS3 proteins, determined by 12% SDS-PAGE, were pooled and dialyzed against Buffer A, first with and then without 2 mM EDTA, concentrated to 10 mg/mL using Centriprep-50 (Millipore), increased to 15% glycerol and stored at −20°C. The yields for wild type and mutant proteins were reproducibly approximately 50 mg of purified protein per liter of *E. coli* culture.

All helicase substrates were composed of annealed complementary RNAs 5′-CACCUCUCUAGAGUCGACCUGCAGGCAUCG-3′ and 3′-GUGGAGAGAUCUCAGC-5′. In double-stranded duplexes, the longer strand was 5′-end labeled with [γ-^32^P] ATP using T4 polynucleotide kinase (PNK, New England Biolab) according to the manufacturer's protocol. The 10-µL reaction mixture (3 µL water, 1 µL 10x PNK buffer, 1 µL RNA or DNA (10 pmol), 4 µL of [γ-^32^P] ATP] (∼ 12 pmol) and 1 µL PNK enzyme) was incubated for 1 hr at 37°C and quenched by heating for 10 min at 95°C. The labeled RNA or DNA was purified by electrophoresis on a 10% acrylamide (30∶1) gel in 1X TBE (89 mM Tris-base, 89 mM glycine, 1 mM EDTA). Regions of the gel containing RNA or DNA were located by autoradiography, sliced from the gel, and incubated for 6 hr at 50°C in 400 µL elution buffer (50 mM Tris-HCl pH 7.0, 500 mM NaCl, 0.1% SDS, 10 mM EDTA) in a thermo-mixer. The gel debris was pelleted by brief centrifugation; the supernatant was treated with 2.5 volumes of cold ethanol and stored overnight at −20°C. The precipitated RNA was collected by centrifugation at 4°C for 30 min. The pellet was washed with 1 mL 70% ethanol, dried and dissolved in 20 µL annealing buffer (25 mM HEPES-KOH pH 6.5 and 50 mM NaCl). The appropriate 5′-end-labeled RNA was mixed at 1∶5 molar ratio with the unlabeled shorter complementary strand and annealed in a thermocycler: 5 min at 95°C, decreased to 60°C at a rate of 1°C/min, 30 min at 60°C, decreased to 25°C at a rate of 1°C/min and incubated overnight at 25°C. The radiolabeled double-stranded substrate was purified by gel electrophoresis as described above (except the elution step was 12 hr at 4°C). The purified duplex was adjusted to a final concentration of 100 fmol/µL in 25 mM HEPES-KOH pH 8.0, 50 mM NaCl and stored in aliquots at −20°C.

### Helicase and ATPase assays

Helicase activity was assayed by 1-hour incubation at 37°C of a 10-µL reaction mixture containing 2.5 mM ATP, 1 mM MgCl_2_, 10 fmol nucleic acid substrate in 25 mM HEPES pH 8.0, 2 mM dithiothreitol, and 5% glycerol as described previously [49]. The standard reaction was initiated by addition of enzyme at the indicated concentration (Fig. 8c) or an equivalent volume of buffer. The reaction was quenched by addition of EDTA to a final concentration of 20 mM and of sodium dodecyl sulfate to a final concentration of 0.1%. Reaction products were separated by electrophoresis on a 15% polyacrylamide native gel and detected using a Typhoon scanner (Molecular Devices).

ATPase activity was assayed in a final volume of 10 µL in a reaction mix containing 50 mM Tris pH 8.0, 10 mM KCl, 1 mM MnCl_2_, 1 µCi [α-^32^P] ATP (3,000 Ci/mmol; Amersham) with or without enzyme, and incubated at room temperature for 10 min. The reaction was quenched by addition of 2.5 µL of 50 mM EDTA. 0.5 µL of the reaction mix was spotted onto a cellulose PEI sheet (J. T. Baker), developed for 30 min by ascending thin-layer chromatography in 0.375 M potassium phosphate pH 3.5, air-dried, and detected using a Typhoon scanner (Molecular Devices).

### Statistical analyses

Analyses of variance (ANOVA) was used to compare mean peak viremia and viral titers at various time point comparisons among NS3-249 mutant viruses for *in vitro* studies (Vero and DEF cell lines) and experimentally infected bird models (AMCRs and HOSPs). Means were compared between WNV NS3-249 mutants using Tukey's HSD adjustment for multiple comparisons. Plaque diameters were compared to the NS3-249P virus by two-sided student t-test.

### Ethics statement

Birds were collected under US Fish and Wildlife Services and Colorado Parks and Wildlife permits with permission of Morning Fresh Dairy as well as the managers of the Colorado State Fisheries Unit in Bellvue, CO. Field studies did not involve endangered or protected species. All animal studies presented herein were approved by Institutional Animal Care and Use Committees at the Division of Vector-Borne Diseases, Centers for Disease Control and Prevention (approval number 13-009), the University of California, Davis (approval number 12874) and Colorado State University (approval number 10-2078A). All protocols and practices for the handling and manipulation of animals (mice, crows and sparrows) were in accordance with the guidelines of the American Veterinary Medical Association (AVMA) for humane treatment of laboratory animals as well as the “Guidelines to the Use of Wild Birds in Research” published by the ornithological council 3^rd^ edition (2010).
